# Chemotaxis and swarming in differentiated HL-60 neutrophil-like cells

**DOI:** 10.1038/s41598-020-78854-6

**Published:** 2021-01-12

**Authors:** Kehinde Adebayo Babatunde, Xiao Wang, Alex Hopke, Nils Lannes, Pierre-Yves Mantel, Daniel Irimia

**Affiliations:** 1 Department of Surgery, BioMEMS Resource Center, Massachusetts General Hospital, Harvard Medical School, Boston, MA USA; 2 Department of Medicine, University of Fribourg, 1700 Fribourg, Switzerland

**Keywords:** Chemotaxis, Biomedical engineering

## Abstract

The human leukemia cell line (HL-60) is an alternative to primary neutrophils in research studies. However, because HL-60 cells proliferate in an incompletely differentiated state, they must undergo differentiation before they acquire the functional properties of neutrophils. Here we provide evidence of swarming and chemotaxis in differentiated HL-60 neutrophil-like cells (dHL-60) using precise microfluidic assays. We found that dimethyl sulfoxide differentiated HL-60 cells (DdHL-60) have a larger size, increased length, and lower ability to squeeze through narrow channels compared to primary neutrophils. They migrate through tapered microfluidic channels slower than primary neutrophils, but faster than HL-60s differentiated by other protocols, e.g., using all-trans retinoic acid. We found that dHL-60 can swarm toward zymosan particle clusters, though they display disorganized migratory patterns and produce swarms of smaller size compared to primary neutrophils.

## Introduction

Neutrophils are immune cells that constitute an integral part of the first line of host defense against infection and tissue injury^1^. Upon pathogenic infection, neutrophils migrate rapidly from peripheral blood through the endothelial cell layer and into tissues, towards the site of infection^2^. In response to infections and tissue injuries, neutrophils often display highly coordinated chemotaxis, accumulation, and a self-amplified activation, i.e.,neutrophil swarming, which is important in neutralizing infections and protecting healthy tissues. A better understanding of neutrophil activities could eventually lead to interventions to enhance neutrophil efficacy against infections and protect tissues during inflammation. However, primary neutrophils are challenging to study directly because of short life span, donor variability, and low transcriptional activity that limits the use of common genetic approaches. Thus, relevant models are needed, and cell line-based models to substitute primary neutrophils would be highly useful.

HL-60 is a commonly used substitute cell line model to study neutrophil phenotypic functions. Several studies have reported the use of HL-60 cells to study migration in neutrophils^3–6^. These studies used assays like micropipette^6^, EZ-TAXIS assay^6^, filter assay^5^, and transwell assay^7^. However, the information provided by the conventional assays is limited. Comparing the differences in migration abilities between HL-60 cells differentiated using different protocols is challenging due to the limited precision of these assays. Moreover, swarming activities in vitro and in vivo have so far only been observed using primary neutrophils^8–10^.

In this study, we employed microfluidic migration assays and a micropatterning array to compare the swarming and migratory ability of HL-60 cells after they were differentiated using DMSO (DdHL-60), all-trans-retinoic acid (AdHL-60) and nutridoma supplemented DMSO (nDdHL-60). We confirmed that nDdHL-60 migrate faster than DdHL-60^11^. We found that both nDdHL-60 and DdHL-60 cells are capable of swarming, suggesting that nDdHL-60 cells are a good model of primary neutrophils. Our study also suggests that differences in migration, deformability, and persistence have to be taken into account when employing differentiated HL-60 cells as models for studying primary neutrophil functions.

## Results

**Figure 1:**
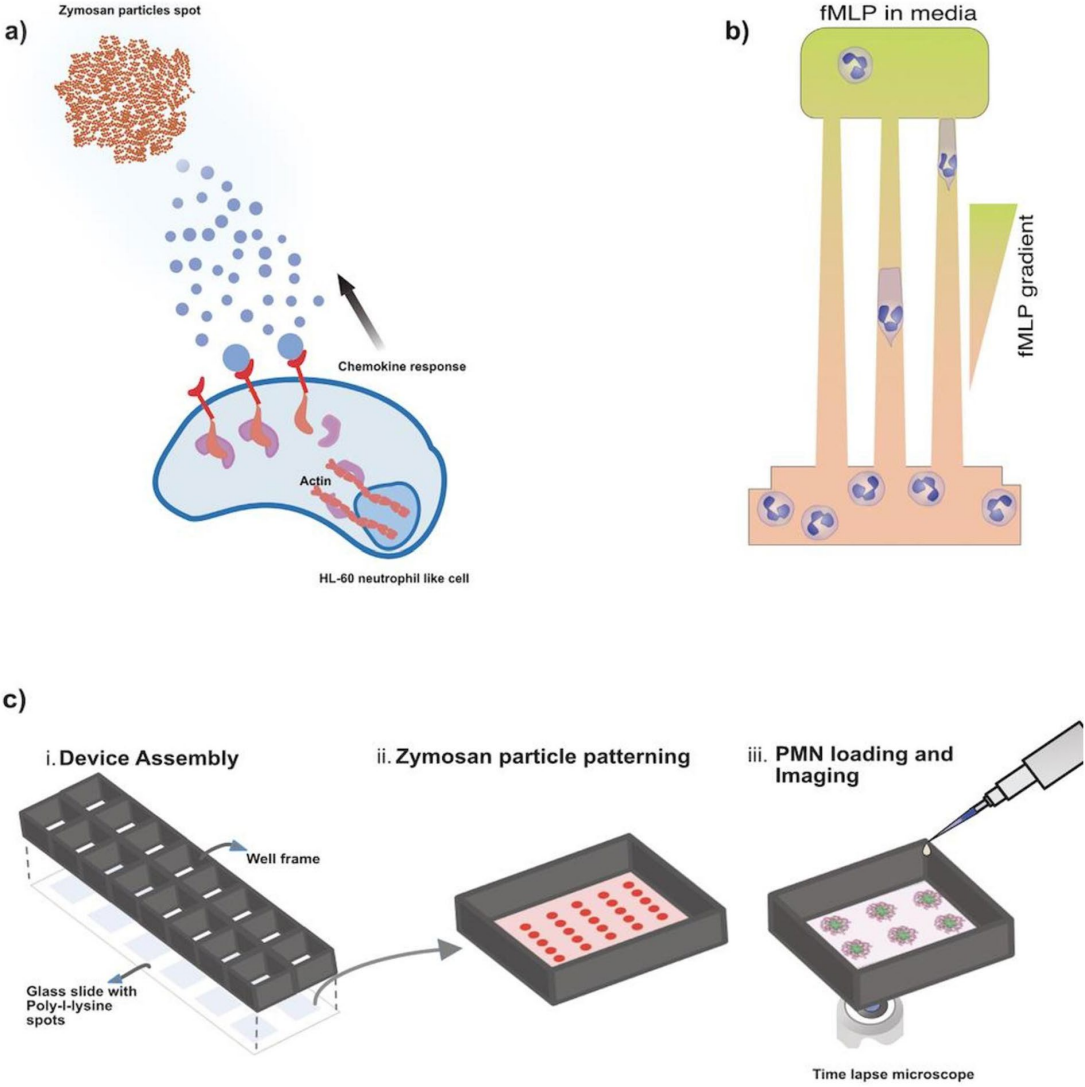
Schematic diagram of tapered channel and swarming assay. (**a**) Schematic image of HL-60 differentiated neutrophil like cells responding to chemokine released by zymosan particles. (**b**) Tapered channel showing neutrophil cells migrating from the cell loading chamber (bottom) to the chambers loaded with chemoattractant fMLP (top). The tapered channel is approximately 500 μm in length and its cross-sectional area decreases from 20 to 6 μm^2^, (**c**) micropatterned zymosan particles (red spots) array for the quantification of HL-60 differentiated neutrophil like cells swarming (i) Schematic illustrations showing the assembly of the 16 well open chamber device, (ii) zoom‐in of one of the zymosan particle patterning in the wells. The size of each zymosan spot is 140 µm^2^ and each spot is 1 mm apart and (iii) subsequent HL-60 differentiated neutrophil like cells loading and imaging. All images were designed by Affinity Designer Software (version 1.8.4.) https://affinity.serif.com/en-us/designer/.

We employed microfluidic devices with arrays of tapered channels to probe the chemotaxis and responses to progressive deformation^12^ and employed patterned zymosan particles clusters to probe the swarming of DdHL-60, nDdHL-60, and primary neutrophils (Fig. 1, Supplementary Video S1).

### Enhanced chemotaxis of DdHL-60 compared to AdHL-60

**Figure 2:**
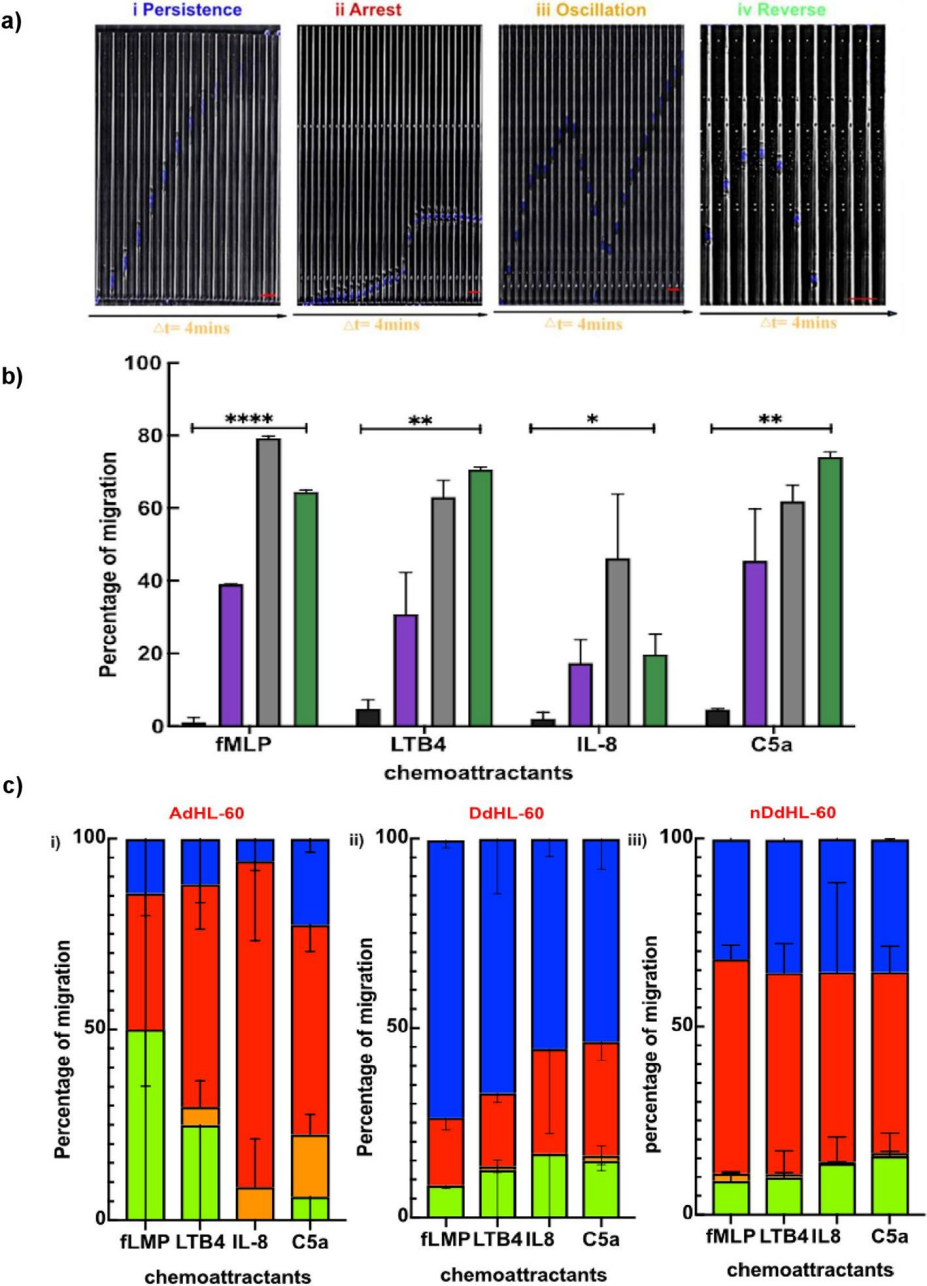
Migration of HL-60 cells through tapered channels after differentiation using different protocols. (**a**) Kymographs show the persistent (P), Arrest (A), Oscillation (O) and reverse (R) migration patterns. The nuclei of the HL-60 cells are stained with Hoechst dye (blue). The time interval between two consecutive frames of the kymographs is 4 min. Scale bar is 25 µm. (**b**) The percentage of migration in fMLP, LTB4, IL-8 and C5a gradients of ATRA (Black bars), DMSO (Violet bars), nDdHL-60 (Grey bars) and primary neutrophils (Green bars) toward each chemokine gradients. (**c**) Percentage of migratory patterns in (i) DdHL-60 cells, (ii) AdHL-60 cells and (iii) nDdHL-60 cells. Persistence (Blue color), Arrest (Red color), Oscillation (Orange color) and Retrotaxis (Green color). Comparison between the percentage of migration towards each chemo-attractants were performed by one-way ANOVA; ****P < 0.0001 in fMLP, **P < 0.01 in LTB4 and C5a and *P < 0.05 in IL-8.

Using tapered-channel devices, we measured the fraction of HL-60 cells migrating and quantified the frequency of various migration patterns (Fig. 2a–c). First, we compared the migratory ability of HL-60 after differentiation using two differentiation protocols. We found that 65%, 39%, and 1% of human neutrophils, DdHL-60, and AdHL-60 migrated in fMLP gradients, respectively (Fig. 2b). The fraction of cells migrating was slightly larger in LTB4 and C5a gradients (71%, 31% and 5% for primary neutrophils, DdHL-60, and AdHL-60, respectively, for LTB4 and 74%, 46% and 5% for primary neutrophils, DdHL-60 and AdHL-60, respectively, for C5a) (Fig. 2b). The fraction of cells migrating in IL-8 gradients was 20%, 17% and 2% for primary neutrophil, DdHL-60, and AdHL-60, respectively (Fig. 2b).

### Enhanced chemotaxis of nDdHL-60 compared to DdHL-60

Nutridoma, a serum-free supplement, has been reported to improve the differentiation of PLB-985 cells while not compromising the viability of the cells^11,13^. To test how serum concentration and nutridoma affect differentiation in HL-60 cells, we checked the chemotactic ability of HL-60 cells upon differentiation in media containing 1.3% DMSO, 2% fetal bovine serum (FBS) and 2% nutridoma. Replacing serum with nutridoma increased the percentage of migrated cells to 79%, 63%, 46%, and 62% in fMLP, LTB4, IL-8, and C5a gradient compared to 39%, 31%, 17%, and 46% for DMSO alone, respectively (Fig. 2b). Together, our data shows that nDdHL-60 cells and primary neutrophils are comparable in terms of migratory ability.

### Differentiated HL-60 cells display distinct migration patterns from primary neutrophils

We compared the migration patterns in dHL-60 cells with those reported before for human neutrophils^12^. We focused on four migration patterns identified in earlier studies, including persistent migration (P), arrest (A), oscillation (O), and retro-taxis (R) (Fig. 2a). We calculated the percentage of cells displaying each migratory pattern and the particularities of responses to each chemokine tested. We found that 73, 66, 63 and 55% of DdHL-60 neutrophils and 25, 12, 30 and 11% of the AdHL-60 neutrophils migrated persistently in fMLP, LTB4, C5a and IL-8 gradients respectively (Fig. 2ci–ii). These numbers were lower compared to 84, 76 and 70% of primary neutrophils migrated persistently in fMLP, LTB4 and IL-8 respectively^12^. In fMLP gradient, 18.5, 7.5, and 0% of the DdHL-60 and 58.5, 24.4 and 4.9% of AdHL-60 neutrophils showed arrest, retro-taxis, and oscillations, respectively (Fig. 2ci–ii). In contrast, 7, 7, and 2% of primary neutrophils showed arrest, retrotaxis, and oscillation in fMLP gradient, respectively^12^. The migration patterns of AdHL-60 included a larger percentage of arrest and retro-taxis compared to DdHL-60 (Fig. 2cii). Surprisingly, the presence of nutridoma in the differentiation media markedly changed the migratory patterns of nDdHL-60 cells, which display frequent arrest and retrotaxis migratory patterns. We observed that 36.4%, 35.4%, 23.6% and 35.2% migrated persistently in fMLP, LTB4, IL-8 and C5a gradient respectively compared to DMSO alone (Fig. 2ciii). In addition, about 65.5%, 64.7%, 76.4% and 64.8% of nDdHL-60 demonstrated more arrest migratory pattern in all chemokine gradient compared to DMSO alone (Fig. 2ciii).

**Figure 3:**
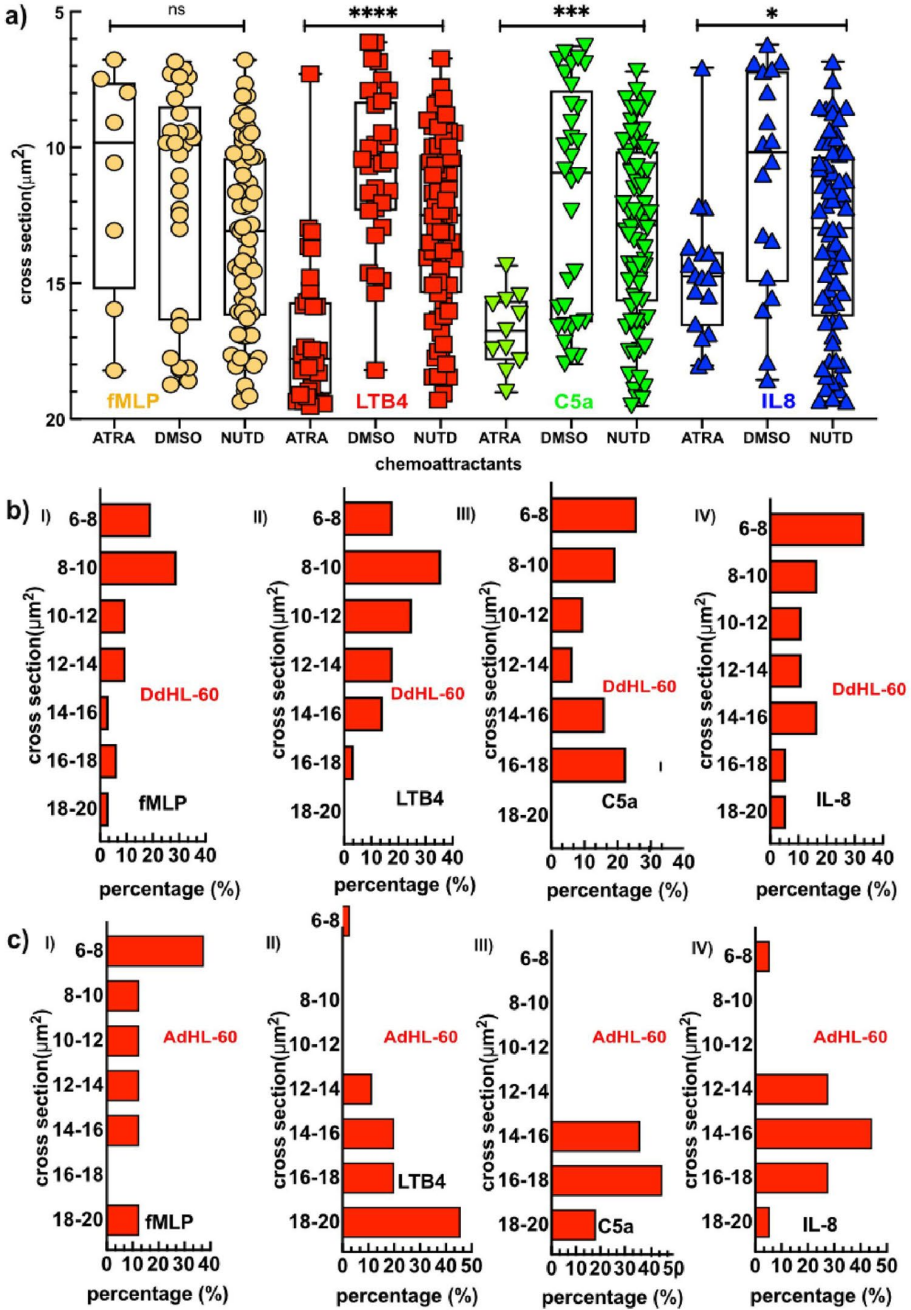
The differentiation protocol alters the ability of HL-60 cells to traverse narrow strictures. (**a**) Critical cross section where Arrest, Oscillation and Retrotaxis occur in (**a**) AdHL-60 (ATRA), (**b**) DdHL-60 (DMSO) and (**c**) nDdHL-60 (NUTD) in tapered straight channel in chemokine gradients of fMLP (Orange), LTB4 (Red), C5a (Green) and IL-8 (Blue). Histograms of Arrest, Oscillation and Retrotaxis critical sections in (**d**) DdHL-60 cells and (**e**) AdHL-60 in different chemokine gradients. Comparison between the number of cells in the different critical cross section toward each chemo-attractants were performed by one-way ANOVA; ****P < 0.0001 in LTB4, ***P < 0.001 in C5a and *P < 0.05 in IL-8.

The tapered microchannels enable specific measurements of the cross-section (CS) at which A, O, and R patterns occur in AdHL-60, DdHL-60 and nDdHL-60 (Fig. 3a,b). Our results demonstrate that the average CSs of AdHL-60 (ATRA) are 10, 18, 17 and 15 µm^2^ in fMLP and LTB4, C5a and IL-8 gradients respectively (Fig. 3a), DdHL-60 (DMSO) are at 10, 10.5, 11 and 10.5 µm^2^ in fMLP and LTB4, C5a and IL-8 gradients respectively (Fig. 3a) and the average CSs of nDdHL-60 (NUTD) are at 13.5, 13.5, 13 and 13.5 µm^2^ in fMLP and LTB4, C5a and IL-8 gradients respectively (Fig. 3a). While the average CSs of primary neutrophils are at 10.5, 9.2 and 10 µm^2^ in fMLP, LTB4 and IL-8 gradient respectively^12^.

The histograms of the critical CS show that a larger percentage of A, O, R patterns in DdHL-60 cells occur at cross-sections smaller than 10 µm^2^ (Fig. 3bi–iv), similar to what was observed in primary neutrophils^12^. The histograms of the critical CS show that A, O, R patterns in AdHL-60 cells are also less dispersed (Fig. 3ci–iv). In summary, our data demonstrate that mechanical constriction in the tapered channel during chemotaxis interfere with the migratory response of dHL-60 to chemokine gradients more than with the migratory responses of human neutrophils.

### dHL-60 and primary neutrophils display distinct morphologies during chemotaxis through confined microfluidic channels

**Figure 4:**
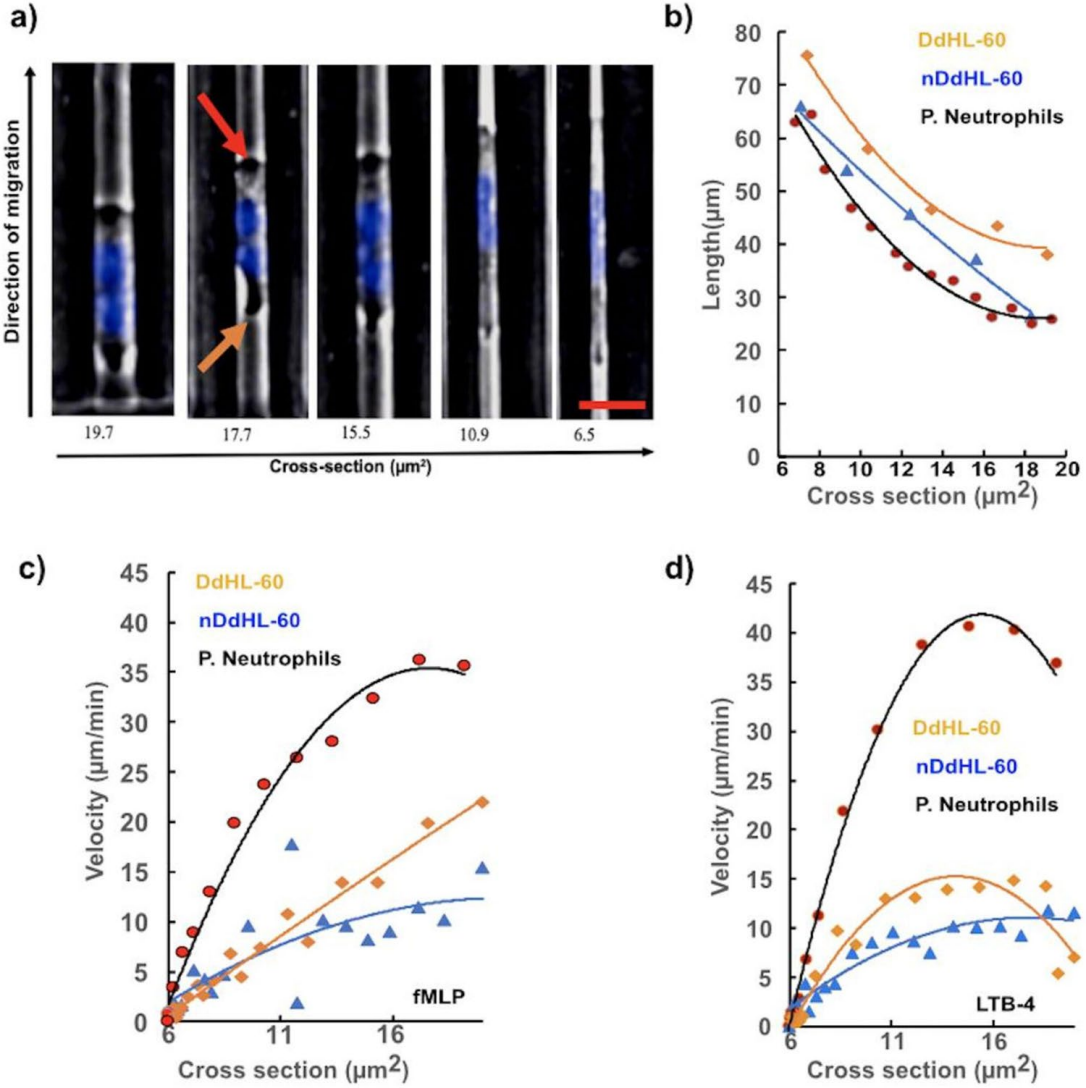
Comparison of cell length, velocity, and velocity changes between dHL-60 cells and primary neutrophils across the different cross-sections of the tapered micro-channel. (**a**) The morphology of dHL-60 cells across changes during the migration through progressively narrow channels. In the larger section of the channels, HL-60 cells display a broad leading edge (Red arrow) and a rounded tail end (uropod) (Orange arrow). The nucleus (blue) of dHL-60 neutrophil deforms to accommodate the migration through the narrowest section of the channels. The leading edge and uropod are no longer distinguishable. HL-60 nucleus is stained with Hoechst dye. The scale bar is 30 µm. (**b**) Change in length across different cross-section of the tapered micro-channel DdHL-60 cells (Orange line), nDdHL-60 cells (Blue line) and primary neutrophil (Black line), (**c**) Change in velocity in fMLP across different cross-section of the tapered micro-channel DdHL-60 cells (Orange line), nDdHL-60 cells (Blue line) and primary neutrophil (Black line). (**d**) Change in velocity in LTB4 across different cross-section of the tapered micro-channel DdHL-60 cells (Orange line), nDdHL-60 cells (Blue line) and primary neutrophil (Black line).

Our data shows that dHL-60 confined in microfluidic channels during chemotaxis are larger and longer than primary neutrophils. We observed that migrating dHL-60 display a broad leading edge toward the chemokine and a rounded tail end (Fig. 4a). When the cells advance through the tapered channels towards the smaller cross sections, their length gradually increases. The length of DdHL-60 cells, nDdHL-60 cells and primary neutrophils increases from ~ 38 to ~ 78 µm, ~ 30 to ~ 65 µm and from ~ 25 to ~ 65 µm respectively when migrating through the tapered micro-channel toward the chemokine (Fig. 4b). We observed that dHL-60 cells migrate slower in all chemokine gradients compared to primary neutrophils. We found that DdHL-60 cells reach 24 (6.6 ± 6.6) and 15 (5.8 ± 6.3) µm/min velocity in fMLP and LTB4 gradients, respectively (n = 10) (Fig. 4c,d). Surprisingly, nDdHL-60 cells are slower compared to DMSO alone as they migrate at a velocity of 15 (4.8 ± 7.3) and 12 (4.5 ± 11.5) µm/min in fMLP and LTB4 gradients, respectively (n = 10) (Fig. 4c,d). The velocities of both DdHL-60 and nDdHL-60 cells in the fMLP gradient decreased with decreasing cross section (Fig. 4c). The highest velocity of DdHL-60 cells was recorded at 13–15 µm^2^ cross-section in LTB4 gradients, while the highest velocity of nDdHL-60 was recorded at 18–20 µm^2^ cross-section in the LTB4 gradients (Fig. 4d). DdHL-60 and nDdHL-60 cells are both slower than primary neutrophils, which can reach velocities as high as 35 (13.6 ± 16.8) and 42 (17 ± 16.8) µm/min in fMLP and LTB4, respectively (n = 10) (Fig. 4c,d). The highest velocity in primary neutrophils was recorded at a cross-sectional area between 16–18 and 14–16 µm^2^ in fMLP and LTB4 gradients, respectively (Fig. 4c,d).

### Reduced directionality of dHL-60 during swarming compared to primary neutrophils

**Figure 5:**
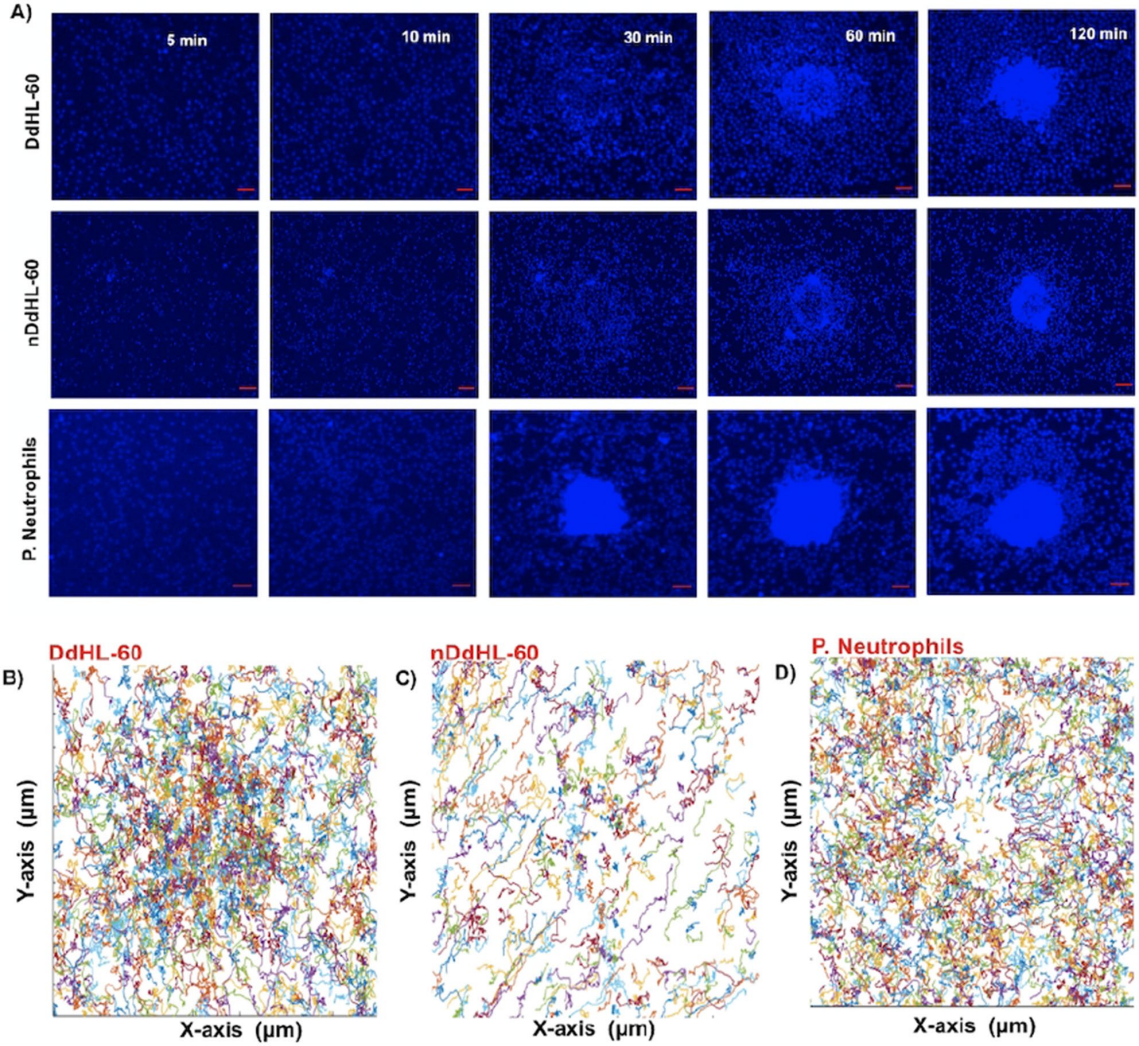
Swarming and aggregation of DdHL-60, nDdHL-60, and primary neutrophils on zymosan-cluster targets. (**a**) Sequential images showing swarm formation in DdHL-60, nDdHL-60 and primary neutrophils (blue) around patterned zymosan particle clusters at 5 min, 10 min, 30 min, 60 min and 120 min (scale bar: 25 µm). Moving (**b**) DdHL-60, (**c**) nDdHL-60, (**d**) primary neutrophils display random and precise track trajectories respectively toward centered zymosan particle. Figure shows representative experiments of N = 4.

Next, we compared the abilities of DdHL-60, nDdHL-60, and human neutrophils to swarm toward zymosan particle clusters. Three distinct phases of swarming have been previously reported^9^. Swarming starts with random migration on the surface (*scouting phase*-5 min Fig. 5a). After the first neutrophil interacts with the cluster, the number of migrating neutrophils towards the zymosan cluster increases rapidly (*growing phase-10–15 min* Fig. 5a). Swarms reach their peak size 60–120 min later, after which the size remains stable (*stabilization phase*-Fig. 5a) (Supplementary video S2). We observed qualitatively similar aggregation dynamics in both DdHL-60 and nDdHL-60 cells. Aggregation starts with the cells moving randomly on the surface of the zymosan particle clusters (*scouting phase*-5 min Fig. 5a). Within minutes of interaction with the zymosan particle, an increasing number of dHL-60 cells migrate in the direction of the zymosan particle clusters (*growing phase-10–30 min *Fig. 5a). A period of fast growth is followed by slower growth. Swarms in nDdHL-60 cells reach their peak size after 60–120 min, after which the size of the aggregates decreases because cells leave the aggregate (Fig. 5a). During the stabilization phase, dHL-60 cells migrate in and out of the swarms (Fig. 5a) (Supplementary video S3 and S4).

**Figure 6:**
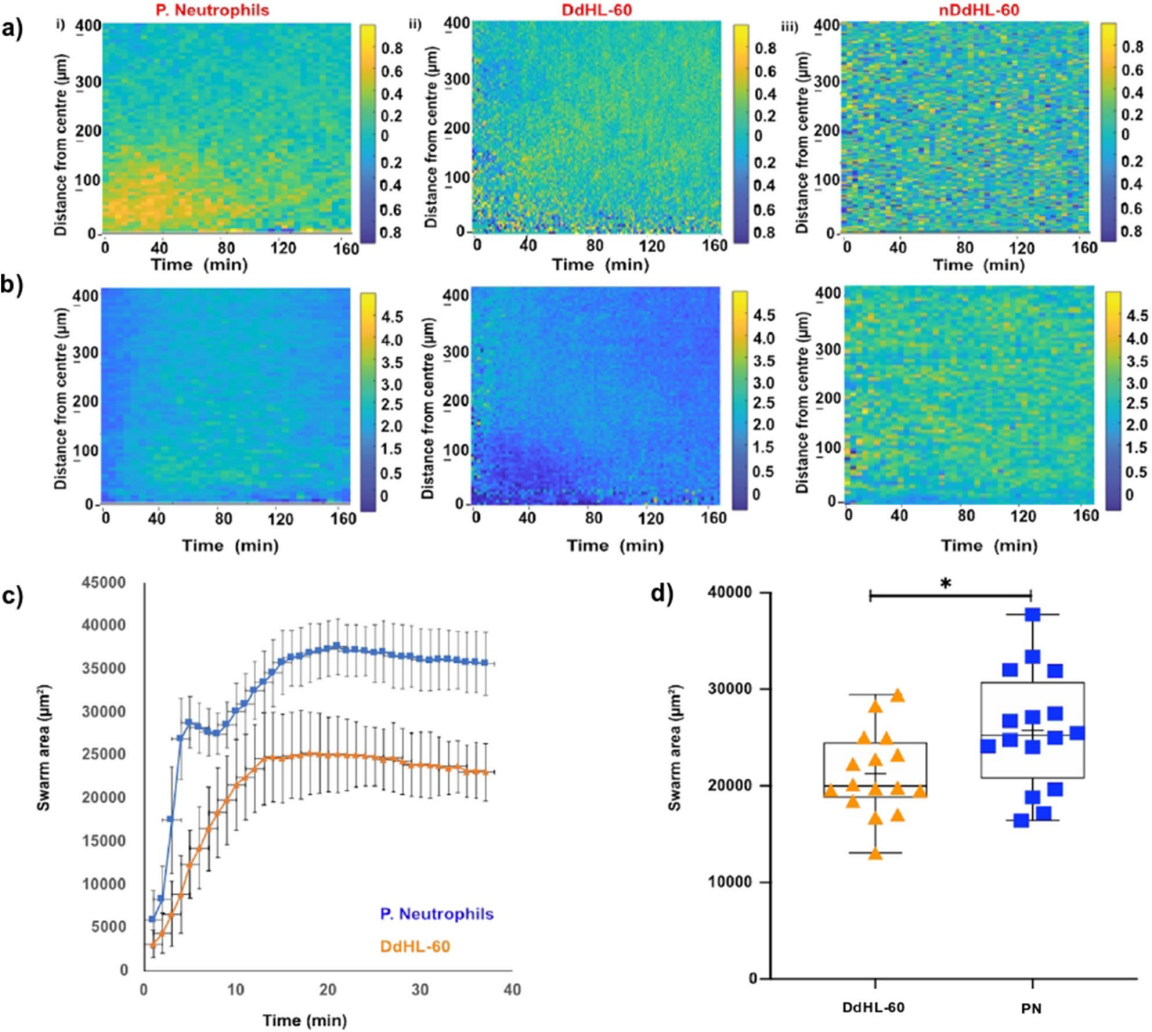
Quantification of primary neutrophil and HL-60 swarming. (**a**) Chemotactic index (CI) and speed over time, towards zymosan particle clusters in (i) primary neutrophils, (ii) DdHL-60, (iii) nDdHL-60. (**b**) The speed of migration increases with time towards zymosan particle clusters (scale bar: CI above 0.8 indicates the cells that are chemotaxing toward zymosan particle. speed (µm/min) = 4.5 means cell moving toward zymosan particle). (**c**) Primary neutrophils (blue curve) accumulation on targets is fast and form a bigger swarm and dHL-60 neutrophil (orange curve) aggregation proceeds fast and then continues slower over time. (**d**) Comparison of area of swarm formed between primary neutrophils (PN) and dHL-60 (HL-60). Comparison were performed by unpaired, two-tailed t-test; *P < 0.05). Error bars represent standard deviations for these measurements. Figure 5 shows representative image of experiment of N = 30 swarm spots.

A quantitative analysis of neutrophil swarming shows significant differences between dHL-60 and human neutrophil swarming. We found that the radial trajectories displayed by dHL-60 cells are more often random and less organized (Fig. 5b,c) compared to swarming primary neutrophils (Figs. 5d, 6ai, Supplementary video S2). Both DdHL-60 and nDdHL-60 cells show increasingly directional migration toward the zymosan clusters at short distances around the zymosan clusters (Fig. 6aii,iii). The migratory speed increases steadily between 10–100 min towards the zymosan particle cluster in primary neutrophils (Fig. 6bi). However, DdHL-60 and nDdHL-60 cells show only slight increase in migratory speed toward zymosan particle cluster (Fig. 6bii,biii). The swarm area around zymosan clusters during the stabilization phase was larger for primary neutrophils compared to DdHL-60 cells, ~ 40,000 μm^2^ vs. 25,000 μm^2^, respectively (Fig. 6c). The mean swarm area around zymosan clusters was ~ 25% larger for primary neutrophils compared to DdHL-60 cell, at 25,000 μm^2^ vs. 20,000 μm^2^, respectively (Fig. 6d).

### Surface expression of LTB4 Receptor 1 and LTB4 secretion by dHL-60 cells

**Figure 7:**
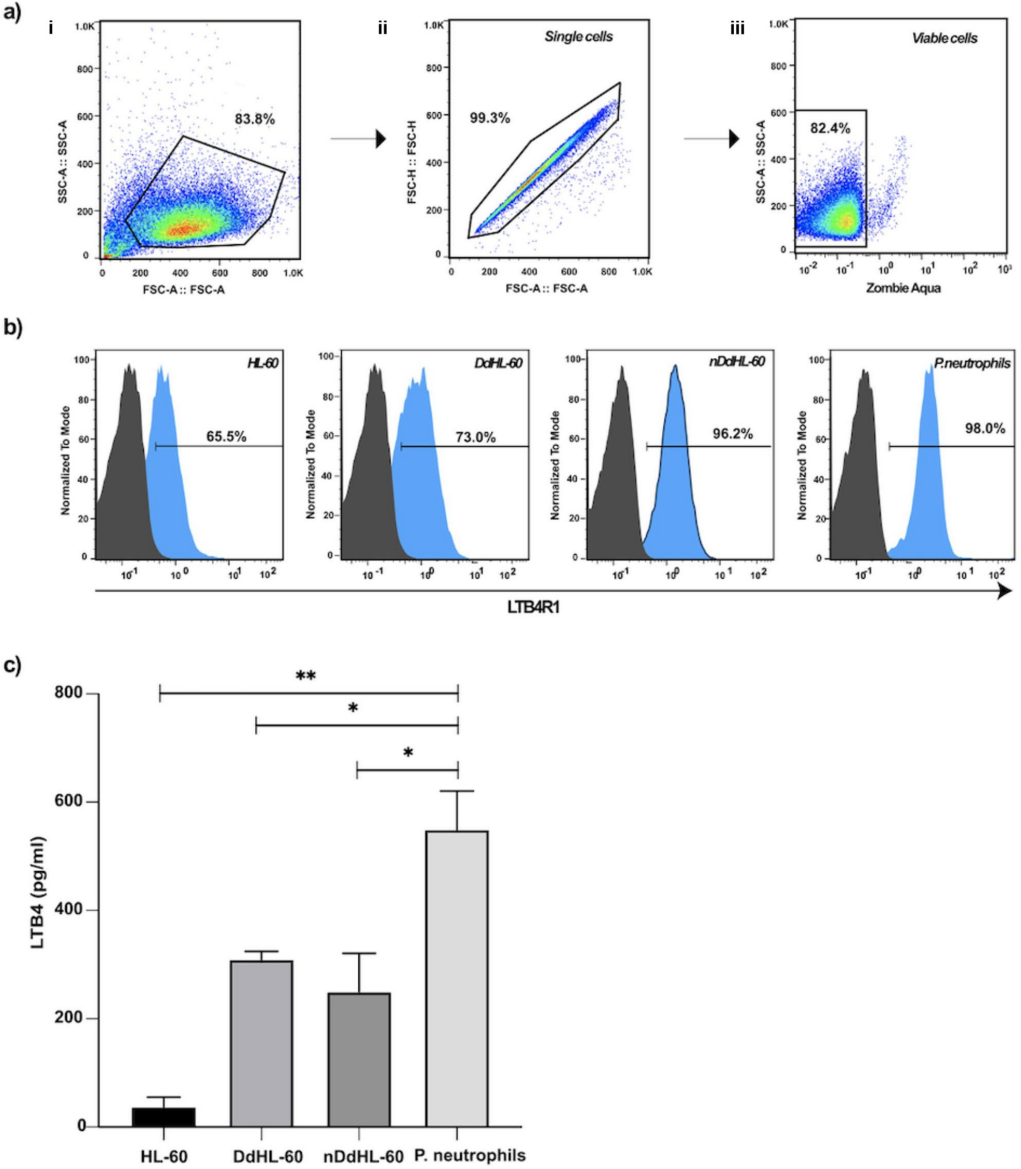
Quantification of LTB4 receptor and release in dHL-60 cells and primary neutrophils. (**a**) Flow cytometry image showing gating strategy of cells based on FSC/SSC profile, subsequently selecting single and viable cells. (**b**) Flow cytometry analysis of expression of LTB4-R1 on gated cells as in (**a**) for undifferentiated HL-60 cells, DdHL-60 cells, nDdHL-60 cells and Primary neutrophils (Black curves: LTB4-R1 negative cells, Blue curves: LTB4-R1 positive cells. Horizontal bars show percentage of LTB4-R1 positive cells), (**c**) HL-60 cells, DdHL-60 cells, nDdHL-60 cells and Primary neutrophils were incubated with *Salmonella typhimurium* for 4 h, and the amount of LTB4 in the supernatant was determined by ELISA (Error bars: mean ± SD; *n* = 3 experiments). Comparison were performed by unpaired, two-tailed t-test; **P < 0.01, *P < 0.05).

Having shown that DdHL-60 cells exhibit swarm like behavior that is qualitatively similar to primary neutrophils but quantitatively distinct, we sought to determine whether the observed differences in DdHL-60 and primary cells was due to expression or secretion of LTB4 and its receptor. To test this, we checked for the expression of LTB4-R1 on viable DdHL-60, nDdHL-60, and primary neutrophils. We found that 73%, 96.2% and 98% of viable DdHL-60, nDdHL-60 and primary neutrophils express LTB4-R1, respectively (Fig. 7a,b). Next, we quantified the amount of LTB4 released during swarming in vitro using enzyme-linked immunosorbent assay (ELISA). For this, we incubated DdHL-60, nDdHL-60, or primary neutrophils with *salmonella typhimurium* for 4 h, then quantified the amount of LTB4 released into the supernatant. We found that the amount of LTB4 released by primary neutrophils was approximately twice the amount released by dHL-60 cells (Fig. 7c).

### Blocking LTB-4 receptors alters DdHL-60 swarm-like behavior

**Figure 8:**
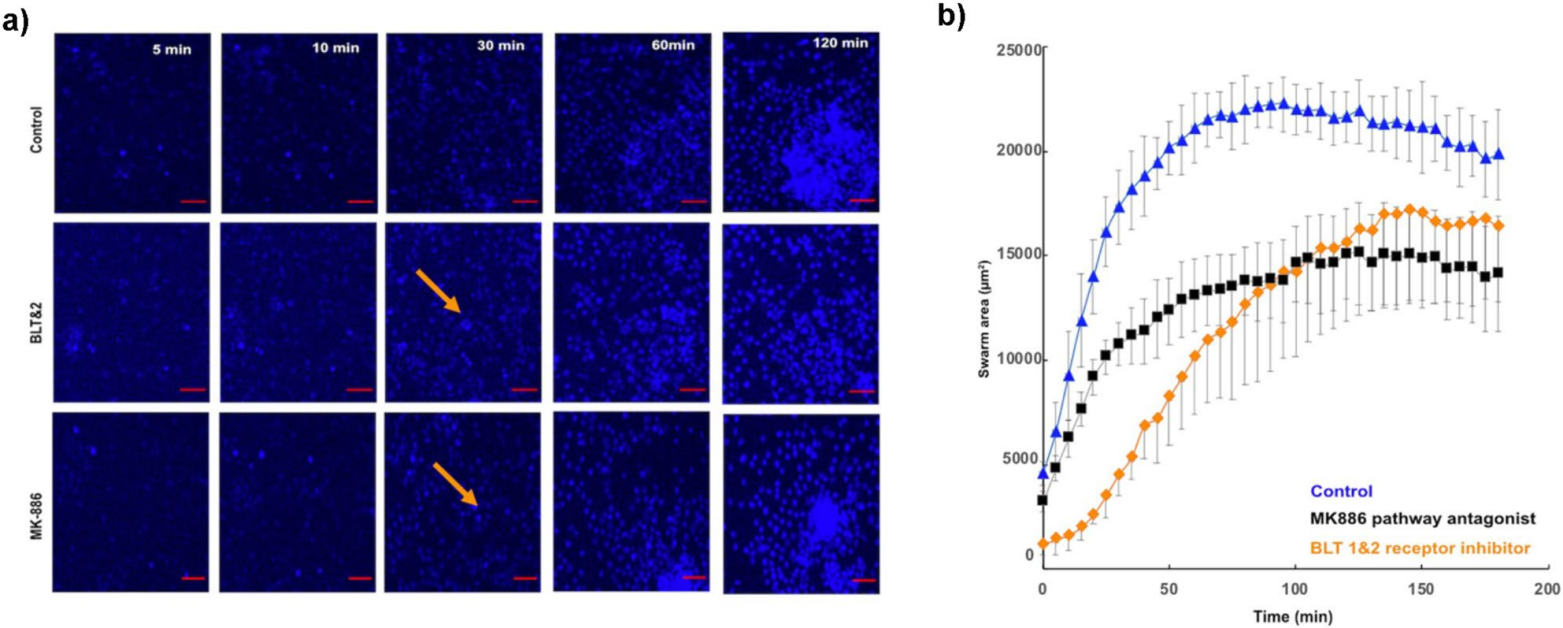
Disrupted migration of DdHL-60 towards swarm in the presence of BLT1 receptor antagonist and LTB4 synthesis inhibitor. (**a**) Sequential images showing swarm formation in Control, BLT1&2 receptor antagonist and LTB4 pathway antagonist around patterned zymosan particle clusters at 5mins, 10mins, 30mins, 60mins and 120 min (scale bar 25 µm). Orange arrows showing delay in initiation. (**b**) dHL-60 form smaller swarms that grow slower in the presence of BLT1 antagonists and LTB4 pathway inhibitors [Blue (triangle) curve: Control, Black (square) curve: LTB4 pathway antagonist MK-886, Orange (diamond) curve: BLT1&2 receptor antagonist]. Each group was measured in four separate spots in a field of view. Error bars represent standard deviations for these measurements. Figure shows representative experiments of N = 36 fields of view (4 spots per field).

To verify that the mechanisms involved in the aggregation of HL-60 cells are similar to swarming in primary neutrophils, we tested the role of LTB4 release during swarming. Previous studies have shown that LTB4 is heavily involved in the positive feedback loop driving the rapid accumulation of primary neutrophils during the growth phases of the swarms^8^. Here, we compared the aggregation of DdHL-60 cells in the presence of BLT1 and BLT2 receptors antagonists and a LTB4 synthesis inhibitor. We found that in the presence of BLT1 and BLT2 receptor antagonists LY255283, U75302 there was significant delay in the initiation and a reduction in the swarm size (Fig. 8a,b) (Supplementary video S6 and S7). Inhibition of the LTB4 pathway by the MK886 inhibitor alters mainly the final size, with little impact on the initiation, suggesting the release of LTB4 during the early stage of swarming is from pre-formed vesicles (Fig. 8a,b) (Supplementary video S6 and S8). The swarm area around zymosan clusters during the stabilization phase was ~ 2 times larger in control compared to drug treated cells (~ 25,000 μm^2^ vs. 17,000 μm^2^ vs. 15,000 μm^2^, for control vs. BLT1&2 vs. MK-886 treated cells) (Fig. 8b).

## Discussion

Neutrophil swarming has been described as a crucial process of neutrophil tissue response needed to specifically regulate tissue protection and destruction during several inflammatory diseases^14^. Here we report that dHL-60 s are capable of swarming, qualitatively similar to primary neutrophils. The dHL-60 s display all three phases of swarming behavior (scouting, amplification, and stabilization phases)^8,9^. However, despite the qualitative similarities, significant differences also exist. The swarm size for similar targets is smaller and the trajectories of cells joining the swarms appear more disorganized for differentiated HL-60 cells compared to primary neutrophils.

The differences in chemotaxis (migration fraction, migratory velocity, and directionality) between DdHL-60, nDdHL-60 and primary neutrophils may contribute to the differences observed in the swarming assay. A lower percentage of DdHL-60 cells migrate towards chemoattractants compared to primary neutrophils and which may be responsible for the observed smaller swarm size in DdHL-60. Furthermore, our data show that nDdHL-60 cells display improved percentage of migration comparable to primary neutrophils, however, with more arrest and retro taxis migratory patterns, which may be the cause of the smaller swarm size observed in nDdHL-60 cells. Moreover, our study also demonstrates that both DdHL-60 and ndDHL-60 cells migrate slower than primary neutrophils^9,10,16^.

Importantly, the third phase of swarming is characterized by the recruitment of distant neutrophils to an infection site, driven by expression of the high-affinity receptor for LTB4 (LTB4R1) on neutrophils^9^. Our data demonstrate that the observed swarm-like behavior in DdHL-60 and nDdHL-60 cells is LTB4 dependent, similar to the findings in primary neutrophils^8^. Though, our data demonstrates that both DdHL-60 and nDdHL-60 cells expresses comparable level of LTB4-R1 to primary neutrophils, but they both release lower amount of LTB4 compared to primary neutrophils during swarming. Since LTB4 release is crucial to the process of swarming in neutrophils, an observed impairment in the release of LTB4 may be the cause of smaller swarm size in DdHL-60 and nDdHL-60 cells.

The establishment of HL-60 cells as a viable model system for neutrophil swarming would have great potential value to the study of swarming. Previously, neutrophil swarming has been demonstrated in zebrafish larvae^15^ and mouse tissues^16^. Human neutrophil swarming has also been observed in vitro^8,17^. Although dHL-60 cells can synthesize LTB4 and also possess LTB4 receptors^3^, to the best of our kowledge, swarming behavior in HL-60 cells has not been demonstrated before.  Compared to these models, HL-60 cells would allow genetic manipulation not possible in primary neutrophils, thereby allowing direct interrogation of the molecular mechanisms at work in swarming without relying on chemical inhibitors with possible off-target effects. Furthermore, HL-60 cells are a long established and relatively easy to use cell line, which would benefit groups for which access to primary human neutrophils is too expensive or difficult.

In summary, our study provides the first evidence for swarming behavior in DdHL-60 cells. The DdHL-60 swarms are smaller than the primary neutrophils even though DdHL-60 s migrate at comparable speed. Differences in the expression of LTB4-R1 on both DdHL-60 and nDdHL-60 cells may explain the differences. Deficits in the mechanisms of intracellular communication or a combination of these factors may also be involved. Our study, therefore, suggests that DdHL-60 and nDdHL-60 could be useful models for the study of neutrophil chemotaxis and swarming, and further optimizations are needed.

## Materials and methods

### HL-60 culture under DMSO and ATRA exposure

HL-60 cells (CCL-240, ATCC, Manassas, VA) were either cultured in RPMI 1640 containing with 10% heat-inactivated fetal bovine serum (FBS), 1% penicillin/streptomycin antibiotics or RPMI 1640 containing 2% FBS, 2% Nutridoma and 5 mL % penicillin/streptomycin antibiotics at 37 °C in 5% CO_2_. Cell cultures were passaged two to three times per week, maintaining cell densities between 1 × 10^6^–2 × 10^6^ cells per ml. Passages were performed in a total volume of 10 mL pre-warmed RPMI 1640 + 10%FBS media, in 75 cm^2^ cell culture flasks. HL-60 cells were differentiated with 1.3% v/v DMSO for 6 days with antibiotics or 5 μM of ATRA for 4 days without media change. For nuclei staining HL-60 neutrophil-like cells were treated with 0.5 μg/ml Hoechst 33,342 (Life Technologies) for 5 min at 37 °C and washed in PBS once.

### Neutrophil isolation

Human blood samples from healthy donors (aged 18 years and older) were purchased from Research Blood Components, LLC. Human neutrophils were isolated within 1 h after the blood draw using a human neutrophil direct isolation kit (STEMcell Technologies, Vancouver, Canada) following the manufacturer’s protocol. After isolation, neutrophils were stained with Hoechst 33,342 trihydrochloride dye (Life Technologies). Stained neutrophils were then suspended in RPMI 1640 media containing 20% FBS (Thermo Fisher Scientific) at a concentration of 2 × 10^7^ cells/ml in the chemotaxis device or 2.5 × 10^6^ cells/mL in the swarming assay.

### Flow cytometry

Cell samples were immunostained with fluorescently conjugated antibodies, BLTR Alexa Fluor 647 (Bio-Rad) and Zombie Aqua Fixable Viability Kit (Biolegend). Samples were run on a multi-colour flow cytometer: MASCQuant (Miltenyi Biotec). Data were analyzed using FlowJo Software (Data analysis Software, Version 10.6.2, Ashland, OR.).

### Gating strategy

Data were analyzed using FlowJo Software. The gating strategy was based on Forward/Side Scatter (FSC/SSC) profile: (i) Cells of interest were obtained by gating on cell population based on size and granularity using (FSC vs SSC ) to exclude debris (Fig. 7ai). (ii) Single cells were identified by gating on cell of interest population by using forward scatter height (FSC-H) versus forward scatter area (FSC-A) density plot for double cells exclusion (Fig. 7aii). (iii) Viable cells were identified by gating on the single cell population negative for Zombie Aqua (Fig. 7aiii).

### LTB_4_ measurement

LTB_4_ was measured using an ELISA kit (R&D Systems Minneapolis, MN). Cell samples at 1 × 10^6^ cells/mL were incubated with *Salmonella typhimurium* in antibiotic containing RPMI culture medium at 37 °C. After incubation, cells were spun down at 400 g for 5 min and supernatants were collected and frozen. Assays were performed according to manufacturer’s instructions.

### Neutrophil inhibition

LY255283, U75302, and MK-886 were dissolved in DMSO. Each compound was re-suspended in cell culture media at a concentration of 20 µM for LY255283 and U75302 and 400 nM for MK-886 pathway inhibitor. For BLT1&2 receptors inhibition, dHL-60 were incubated in LY255283, U75302 for 30 min and for LTB4 synthesis inhibition, dHL-60 were incubated in MK-886 for 30 min before adding on the swarming assay.

### Device fabrication

The microfluidic devices were fabricated as described by Wang and colleagues using standard soft lithography^12^. Briefly, two-layer master mold in negative photoresist (SU-8, Microchem, Newton, MA) were fabricated on a 4-inch silicon wafer. The first layer was 2 μm thin containing the patterns of the tapered migration channels. The second layer was 75 μm thick and consists of cell-loading channels (CLC) and chemokine chambers. A ratio of 10:1 PDMS base and curing agent were mixed, cast on the master mold, and degassed thoroughly (PDMS, Sylgard, 184, Elsworth Adhesives, Wilmington, MA). We transferred the wafer into an oven at 65**◦**C to cure overnight. After curing, we peeled off the PDMS layer from the wafer and cut out individual devices using a scalpel. We punched the inlets and outlets of the devices using a 0.75 mm diameter biopsy puncher (Harris Uni-Core, Ted Pella) and irreversibly bonded them to a glass-bottom multi-well plate (MatTek Co., Ashland, MA). To prepare the glass slides for the swarming assay, plasma treated glass slides (Fisher brand Double Frosted Microscope Slides, Fisher Scientific, Waltham, MA, USA) were micro-patterned with a solution containing poly-L-lysin and FITC-ZETAG (1.6 mg/ml) using a Polypico micro-dispensing machine^17,18^. Zymosan particle clusters were used as targets for neutrophils swarms. A solution of 0.5 mg/mL zymosan particles in ultra-pure water (Gibco, life technologies, USA) was prepared and sonicated for 10 min before pipetting onto the glass slide and was allowed to adhere for 10mins on a hot plate. Excess zymosan particles was then washed thrice with PBS and stored at room temperature. Prior to the experiment, glass slides were placed in an open well chamber (Grace Bio-Labs). For HL-60 neutrophil-like cells, the chambers were coated with 50 μg/ml of fibronectin for 1 h at 37 °C to improve migration of the cells.

### Microfluidic devices to study neutrophil chemotaxis

The microfluidic device used for this study was designed as described by^12^ and it consists of an array of tapered channels, with a cross-sectional area of 20 μm^2^ at the cell loading chamber end to 6 μm^2^ at the chemo-attractant chamber end. The tapered channels are 500 μm in length and connect the cell-loading chamber (CLC) to several chemoattractant chambers. A chemoattractant gradient that increases toward the chemoattractant chamber is established along the tapered migration channels. This device enables us to compare the chemotaxis and migration between HL-60 cell line model of neutrophils and primary neutrophils. Using time-lapse imaging and automatic cell tracking on image J software, we were able to effectively compare the active migration patterns between a single DdHL-60 neutrophil-like cell and a single primary neutrophil with high spatial and temporal resolutions.

### Device set-up and cell loading

fMLP (Sigma-Aldrich), LTB4 (Cayman Chemical, Ann Arbor, Michigan, USA), C5a and IL-8 (Cayman Chemical, Ann Arbor, Michigan, USA) were diluted in RPMI to a working concentration of 100 nM. C5a was diluted in RPMI without FBS to working concentrations of 100 nM. Five microliters of the chemokine solution were pipetted into each device. The well plate was then placed in a desiccator under vacuum for 15 min. The well plate was then taken out from the desiccator for 15 min until the devices were filled with the solution. Three milliliters of media (RPMI + 10% FBS) was then added to each well to cover the devices. Each device was then washed by pipetting 10 μl media through the inlet. Finally, about 5 × 10^4^ HL-60 neutrophil-like cells were pipetted into each device. Time-lapse images of neutrophil migration were captured at 10°ø magnification using a fully automated Nikon TiE microscope (Micro Device Instruments). The microscope is equipped with a bio chamber heated at 37**◦**C and 5% CO2.

### Analysis of differentiated HL-60 neutrophil-like cells (dHL-60) migration

We used Track-mate module in Fiji ImageJ (ImageJ, NIH, version 2.0.0) to track and analyze cell trajectories automatically. We identified four migration behaviors, including persistent migration (P), arrest (A), oscillation (O), and retro-taxis (R). Persistent migration indicates neutrophils that migrated through the tapered channels without changing directions from the cell loading chamber to the chemoattractant chamber. Arrest describes neutrophils that migrated into the tapered channels but got trapped in the channels. Oscillation indicates neutrophils that change migration direction more than two times within the tapered channel. Retro-taxis describes neutrophils that migrated into the tapered channel but migrated back into the cell-loading chamber. Percentage of migration was calculated as thus: (N_mc_)**/**(N_CLC_)*100. Where N_mc_ is total number of migrated cells in the tapered channel and N_CLC_ is total number of cells in the cell loading chamber. Percentage of each migration pattern was calculated as thus: (N_mp_)**/**(N_mc_)*100. Where N_mp_ is number of cells demonstrating a specific migration pattern and N_mc_ is total number of migrated cells in the tapered channel.

### Swarm size measurement

Changes in swarm size over time were estimated using track mate plugin in Image J. The cell-occupied area was measured from the DAPI channel for Hoechst labeled HL-60 neutrophil-like cells and primary neutrophil using filter and suitable threshold on image J^8^.

### Chemotaxis and tracking analysis

To quantify the directional radial migration during swarming, we measured the distance of neutrophils to the zymosan spot and plotted the changes in this distance for individual tracks over time. The instantaneous chemotactic index (CI) at time *t* was then calculated as: CI(t) =  − R′(t)/X′(t) where: R′(t) = ∂/∂t ‖x − z‖‖ is the rate of change of the distance between the cell’s position *x* and the zymosan particle cluster position *z*. Before chemotaxis, analysis the cell track positions *x* was determined using track mate plugin in Image J. Clusters of zymosan particles were segmented by Huang thresholding followed by selection of the largest connected component, and *z* was set to be the centroid of this object. Cellular migration speed (spline smoothed) is calculated as: X′(t) = ‖∂x/∂t‖.

### Statistical analysis

Statistical significance of the differences between multiple data groups were tested using two-way Analysis of Variance (ANOVA) in GraphPad Prism (GraphPad Software, version 8.3.0). Within ANOVA, significance between two sets of data was further analyzed using two-tailed **t**-tests. All the box plots consist of a median line, the mean value (the cross), and whiskers from minimum value to maximum value.

## Supplementary information

Supplementary video 1.

Supplementary video 2.

Supplementary video 3.

Supplementary video 4.

Supplementary video 5.

Supplementary video 6.

Supplementary video 7.

Supplementary video 8.

Supplementary captions.
